# Examination of the Liver

**Published:** 1890-04-26

**Authors:** 


### SURGERY.

### EXAMINATION OF THE LIVER.

In an article on the surgical aspect of hepatic abscess in the *B. M. J.*, Mr. Rickman J. Godlee, surgeon to University College Hospital, says something worth repeating on the anatomy of the liver. He remarks: "How different is the liver of to-day from the liver of our studentship! Then it was a flat body with two planes, or at most somewhat uneven surfaces, and a circumference, the latter being broad and thick behind, but thin in front, and to the left." Now, we recognise three well-marked surfaces, and we know exactly what fits into each of the numerous and well-marked impressions. As of old, the upper surface is closely applied to the diaphragm, and for a very small space both the right and left lobes are in contact with the abdominal wall at the epigastrium; but we are able to make out a distinct impression close to the crossing of the suspensory and coronary ligaments, corresponding to the under-surface of the heart. The Spigelian lobe is banished to the posterior surface, and no longer bounds the foramen of Winslow, this function being fulfilled by the Caudate lobe; but the Spigelian lobe rests against the bodies of the tenth and eleventh dorsal vertebræ, and the right cruss of the diaphragm and the aorta. Next, to the right, comes the vena cava in its special fossa, while still further to the right is the space between the two layers of the right lateral ligament, which touches the diaphragm, opposite the tenth and eleventh ribs, and has a distinct little impression for the supra-renal capsule. Here, that is, at the tenth interspace, we might puncture the liver without wounding the peritoneum, but not without traversing the pleura and the lung. The under aspect of the left lobe has a beautifully-curved surface corresponding to the upper half of the anterior portion of the stomach, including the pylorus, which should touch the neck of the gall bladder. Then comes the gall bladder in its own special fossa, and just to the right of its neck is a well-marked pit for the first part of the duodenum. Still further to the right are two obvious fossæ separated by a pretty sharp ridge: the anterior reaches the free border of the gland and receives the hepatic flexure of the colon, the posterior reaches the posterior surface and is occupied by the kidney. The longest axis of the liver is directed from behind forwards, and to the left of the lower border corresponds nearly with the margin of the ribs behind, and as far forward as the ninth cartilage, and then it stretches across ascending somewhat to reach the left eighth cartilage. The upper limit is marked on the surface of the chest by a line drawn from the fifth right to the sixth left chondro-sternal articulation, but varies, as does the lower border, somewhat with inspiration and expiration. The right lung lies in front of the liver, as far down as the upper border of the sixth rib in the nipple line, and the heart as far down as the fifth interspace on the left side. It extends, as a rule, 1½ to 2 inches to the left of the left border of the sternum. Now the above is the best description of the position and anatomical relations of the liver which has ever been written, and the recognition of the facts so lucidly set forth by Mr. Godlee, is all important in coming to an understanding as to the method of rupture of a hepatic abscess. But the liver is not such an immoveable organ as Mr. Godlee's description would lead us to suppose. Varieties of position often occur. During utero-gestation the liver is often pressed considerably above its plane. In its natural position the thin margin of the liver scarcely reaches the border of the thorax, but in females who have laced tightly the liver may be forced several inches below the base of the thorax. In such cases the direction of the aspects of the organ must be changed, and the relation with surrounding parts must be altered. The liver will also change its position somewhat when a person lies on the left side. People who are in the habit of riding much, especially on camels, usually present a liver coming lower down than others who do not take such exercise. Several cases have been recorded of the liver being found on the left side of the body instead of the right; varieties of form also occur. The left lobe is sometimes very small, and has been found only connected with the right by a thin narrow band of tissue. In females who have laced tightly the liver may be marked by pressure of the ribs. Instead of the normal number of lobes, the liver has been found divided into several more. Practically these alterations in position and form are not vitally important. But it is as well to recollect what has been said with regard to effect on position of the liver of camel-riding especially. It may also be usefully recollected that a full stomach and a distended colon push the liver backwards. Endeavours have been made to correspond the area of hepatic dulness with the height of the individual, but no reliable results have been obtained. As a matter of fact, some persons have naturally smaller livers than others, and some larger, and this without disease. Therefore the area of hepatic dulness, as given in text-books, must, while frequently correct, be always accepted as approximate only.

